# War Emergency Medicines

**Published:** 1917-11-17

**Authors:** 


### WAR EMERGENCY MEDICINES.

Since the General Medical Council in July of this year withdrew from the British Pharmacopœia the preparations containing sugar and glycerine no standards have been laid down to replace these preparations, and consequently there has been no uniform system for the dispensing of sugar- and glycerine-containing compounds. On December 1 this omission will be rectified, for on that date a new set of standards will come into operation, the Pharmaceutical Society, in consultation and by agreement with the British Medical Association, having prepared a series of formulæ for sugarless and glycerineless compounds corresponding to those which formerly contained sugar or glycerine. These new formulæ are published as an addendum to the British Pharmaceutical Codex. National Health Insurance practitioners will prescribe according to these formulæ, and it is expected that other medical practitioners will do the same as far as possible. There are, perhaps, circumstances in which medicines containing either sugar or glycerine are essential in the treatment of patients; but where neither one nor the other is essential it is of national importance that neither of them should be prescribed, since all the glycerine that can be produced is required for war purposes, while, as everybody knows, our inadequate supplies of sugar must be conserved.

The War Emergency Formulary here referred to contains a hundred and thirty formulæ, for it comprises not only substitutes for Pharmacopœial preparations but for other sugar and glycerine compounds that are commonly prescribed. It has not been possible to select a substitute for simple syrup that will be suitable in all cases, but, generally speaking, the substitutes used are either diluted glucose (90 parts of glucose and 10 parts of distilled water) or an artificial syrup known by the appropriate name of "Syrupus Factitius." The formula for artificial syrup is as follows: Tragacanth, 0.7; chloroform, 0.5; distilled water, to 100.0. Clearly this is a poor substitute as a sweetening agent, but otherwise it serves its purpose fairly well.

In solid preparations various devices have been adopted; thus confection of senna contains glucose instead of sugar, and in compound liquorice powder the sugar is replaced by rice or maize starch—a substitute which brings the preparation down to the required strength but does not produce a palatable compound. As regards glycerine preparations, the difficulty has been overcome in some cases by the simple process of omitting the glycerine, and in others by substituting artificial syrup or by the use of a small quantity of alcohol. For instance, glycerine of tannic acid is made with tannic acid, tragacanth, chloroform, alcohol, and distilled water; this is one of the few preparations the strength of which has been diminished, for the reason that it is more readily absorbed than when made with glycerine. The glycerine in compound tincture of cardamoms, compound tincture of senna, and tincture of rhubarb has been replaced by a small additional quantity of alcohol.

It will not be necessary for prescribers to concern themselves about the names of the new preparations we have referred to, as in every case the original title has been retained, and the doses remain approximately the same. It will suffice if the prescriber merely adds the letters "W.E.F." (War Emergency Formulary) to the name of the preparation. But since it has been impracticable in many cases in which glucose and artificial syrup replace glycerine or sugar in a formula to preserve all the physical properties of the original preparation as regards either appearance or taste, it will be advisable for practitioners to explain to their patients that any difference they may notice in their medicine is due to a war-time exchange of adjuvants or other components which in no degree impairs the activity or efficiency of the medicine. This refers, of course, to old prescriptions which the practitioner directs to be repeated. In the case of new prescriptions it will not be necessary for the prescriber to give any explanation, as the medicine dispensed according to the prescription should be uniform throughout the country.

